# QUest for the Arrhythmogenic Substrate of Atrial fibRillation in Patients Undergoing Cardiac Surgery (QUASAR Study): Rationale and Design

**DOI:** 10.1007/s12265-016-9685-1

**Published:** 2016-03-02

**Authors:** Lisette J. M. E. van der Does, Ameeta Yaksh, Charles Kik, Paul Knops, Eva A. H. Lanters, Christophe P. Teuwen, Frans B. S. Oei, Pieter C. van de Woestijne, Jos A. Bekkers, Ad J. J. C. Bogers, Maurits A. Allessie, Natasja M. S. de Groot

**Affiliations:** Translational Electrophysiology, Department of Cardiology, Erasmus Medical Center, Thorax Center, PO Box 2040, s Gravendijkwal 230, 3015 CE Rotterdam, The Netherlands; Department of Cardiothoracic Surgery, Erasmus Medical Center, Rotterdam, The Netherlands; Department of Physiology, Cardiovascular Research Institute Maastricht, Maastricht, The Netherlands

**Keywords:** Atrial fibrillation, Epicardial mapping, Electrophysiology, Cardiac surgery, Study design

## Abstract

The heterogeneous presentation and progression of atrial fibrillation (AF) implicate the existence of different pathophysiological processes. Individualized diagnosis and therapy of the arrhythmogenic substrate underlying AF may be required to improve treatment outcomes. Therefore, this single-center study aims to identify the arrhythmogenic areas underlying AF by intra-operative, high-resolution, multi-site epicardial mapping in 600 patients with different heart diseases. Participants are divided into 12 groups according to the underlying heart diseases and presence of prior AF episodes. Mapping is performed with a 192-electrode array for 5–10 s during sinus rhythm and (induced) AF of the entire atrial surface. Local activation times are converted into activation and wave maps from which various electrophysiological parameters are derived. Postoperative cardiac rhythm registrations and a 5-year follow-up will show the incidence of postoperative and persistent AF. This project provides the first step in the development of a tool for individual AF diagnosis and treatment.

**Electronic supplementary material:**

The online version of this article (doi:10.1007/s12265-016-9685-1) contains supplementary material, which is available to authorized users.

## Introduction

Atrial fibrillation (AF) is characterized by beat-to-beat changes in the pattern of activation within the atria, unlike organized arrhythmias such as atrial flutter and atrial tachycardia. This chaotic nature poses a challenge with regard to understanding the pathophysiology and effective treatment of AF as shown by the frequent recurrences after AF therapy [1–4]. Due to the limited knowledge about the mechanisms involved, each AF patient is currently approached in the same manner. Based on the symptomatology and a clinical assessment the arrhythmia is either accepted or attempts are made to retain sinus rhythm with non-selective treatment modalities. However, this approach does not take account of the diversity among AF patients. AF occurs, for example, in association with mitral valve disease, hypertension, congenital heart disease, or cardiac surgery, or in young or older patients without any comorbidity (“lone AF”) [5, 6]. Furthermore, AF can have different clinical manifestations including paroxysmal, persistent, or longstanding persistent. On the structural level, the degree of fibrotic tissue in AF patients demonstrated heterogeneity as well and does not always predict the severity of the AF burden [7]. Therefore, it is likely that the pathophysiological mechanisms may differ between patients with AF. If these can be unraveled the possibility for targeted treatments may arise.

So far, several ablation procedures have been developed aiming to ablate a trigger site for initiation of AF or an arrhythmogenic substrate perpetuating AF. The isolation of triggers residing in the pulmonary veins demonstrated to be most successful in patients with paroxysmal AF. Nonetheless, recurrences occur frequently especially in patients with persistent AF, suggesting an incomplete eradication, reformation, or progression of the arrhythmogenic substrate. Other strategies include the ablation of rotors, ganglionated plexi, and complex fractionated electrograms [8–10]. However, these therapies have similar, limited success rates and there are no guidelines as to which strategy to choose for an individual patient.

The present study has been designed to identify the arrhythmogenic substrate in individual AF patients with the use of a high-resolution epicardial mapping approach. In previous studies, high-resolution epicardial mapping of patients with Wolf-Parkinson-White syndrome or longstanding persistent AF demonstrated to be a valuable tool in discriminating between patients [11, 12]. However, mapping was performed at only three locations and in a limited number of patients with a variety of heart disorders. In this study, subjects are categorized according to the underlying heart disorder(s) and predisposition for developing spontaneous episodes of AF before or after cardiac surgery and epicardial mapping will be performed of the entire epicardial surface [13, 14]. The electrophysiological properties of the atria will be analyzed aiming to find the arrhythmogenic substrate and to contribute to the current knowledge of the pathophysiology of AF.

## Methods

### Study Population

**Fig. 1:**
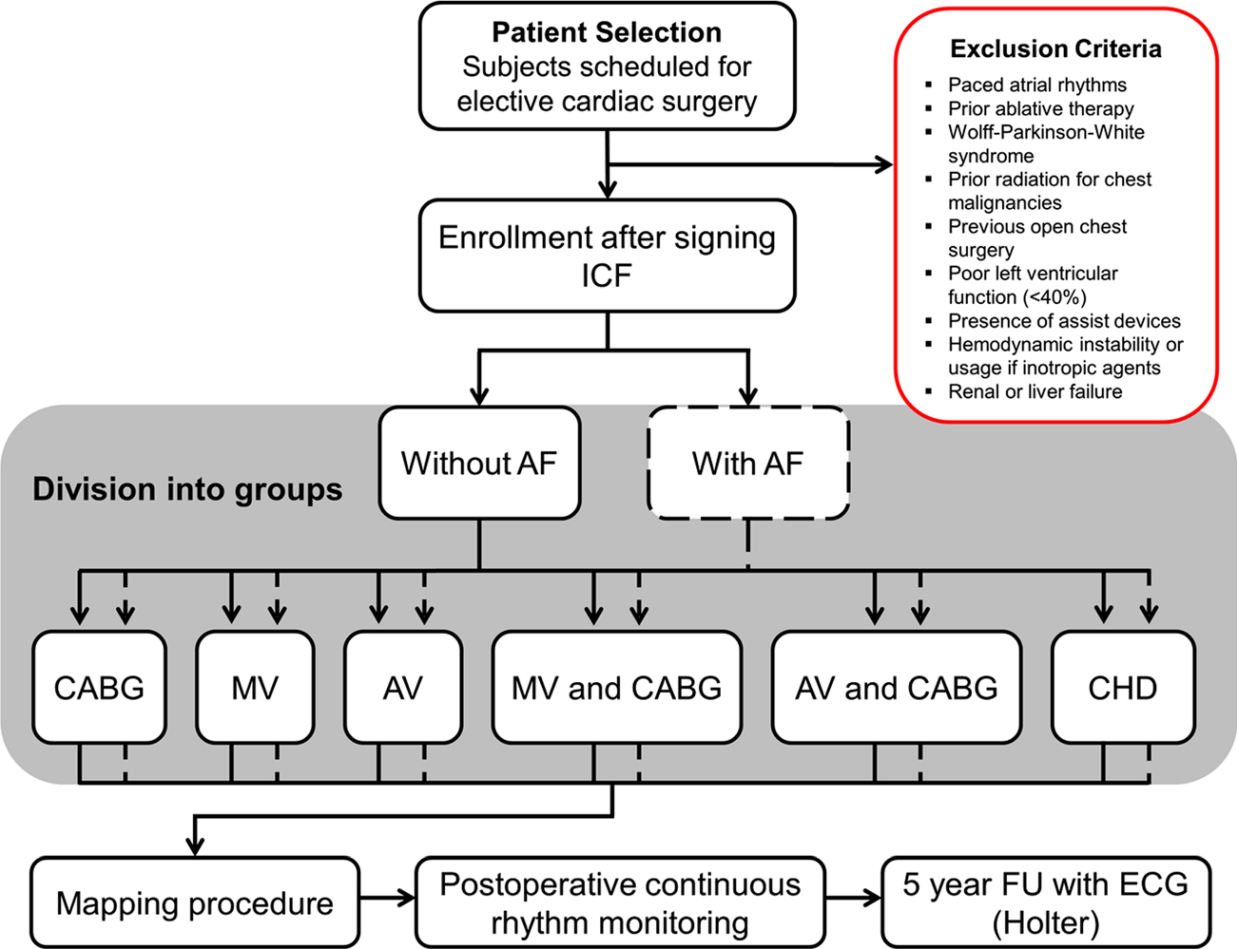
Flow-chart of patient inclusion and following study procedures. After enrollment, patients are assigned to 1 of 12 groups for data analysis according to the presence of previous atrial fibrillation (*AF*) occurrence and the type of surgery that will be performed (i.e., underlying heart disease). Subsequently, all patients are mapped during surgery and continuously monitored after surgery to detect postoperative AF. During the 5-year follow-up (*FU*), the additional tests consist of an electrocardiogram (*ECG*) and Holter monitoring when patients indicate symptoms suspected of AF. *ICF* informed consent form, *CABG* coronary artery bypass grafting, *MV* mitral valve surgery, *AV* aortic valve surgery, *CHD* congenital heart disease

All patients 18 years and older, with structural or coronary heart disease scheduled for elective cardiac surgery will be asked to participate. Patients who have a high-risk of complications during surgery or hemodynamic instability by inducing AF such as Wolff-Parkinson-White syndrome, poor left ventricular function (<40 %), presence of assist devices, hemodynamic instability, usage of inotropic agents, and kidney or liver failure are excluded from this study. Furthermore, patients with medical histories predisposing them for adhesions making epicardial mapping unfeasible or presence of an iatrogenically altered atrial electrophysiology such as prior radiation of the chest for malignancies, redo-cardiac surgery, paced atrial rhythms, and prior ablative therapy in the atria are excluded as well. Each patient, prior to enrolling in the study, will be provided with a written explanation of the study procedure together with an assessment of risks in participating in the study. Patients will be enrolled after the written informed consent form is signed. After enrollment patients are assigned to a group according to the underlying heart disease and whether their medical history includes AF. These groups consist of the following surgical procedures: coronary artery bypass grafting (CABG), mitral valve surgery, aortic valve surgery, mitral valve surgery with CABG, aortic valve surgery with CABG, and congenital heart surgery. Each of these groups are divided into separate groups for patients with and without prior AF episodes. Figure 1 demonstrates the inclusion and following procedures for patients participating in the study.

### Study Procedure

Epicardial mapping is performed during open heart surgery [13]. Patients will be under general anesthesia and vital signs will be monitored continuously throughout the procedure. Mapping will be performed before going on extracorporeal circulation, during sinus rhythm, and (induced) AF. AF is induced by fixed rate pacing at the right atrial appendage with a pulse width of 2 ms delivered by temporary pacemaker wires. Pacing bursts will start at a rate of 250 bpm and will be increased with steps of 50 bpm each time AF is not induced after 3 attempts. If AF is not induced at a pacing rate of 400 bpm or loss of capture occurs, attempts will be terminated. As AF is induced it may terminate spontaneously, otherwise, if an induced arrhythmia sustains after the mapping procedure, electrical cardioversion will be performed immediately afterwards. If a patient is in AF at the onset of the mapping procedure, mapping will be performed during AF and during sinus rhythm after electrical cardioversion if there is no atrial thrombus present on transesophageal echocardiogram.

Epicardial mapping of the right and left atria will be performed using a custom-made electrode array (192 electrodes, diameter 0.45 mm, 2-mm inter-electrode distance; GS Swiss, Küssnacht, Swiss). All electrograms recorded by the electrode are stored on hard disk after amplification (gain 1000), filtering (bandwidth 0.5–400 Hz), sampling (1 KHz) and analogue to digital conversion (16 bits). An indifferent electrode is attached to a steal wire fixed in subcutaneous tissue and a reference signal is attached to the right atrium. In addition, a ventricular surface electrocardiogram (ECG) is recorded simultaneously. Signals will be recorded at 9 right and left atrial sites during sinus rhythm for 5 s per site and during (induced) AF for 10 s per site. Mapping is initiated at the lower right atrium and is proceeded upwards over the right atrial appendage. Thereafter, the left atrium will be mapped starting between the pulmonary veins and will continue along the atrioventricular groove from the lower pulmonary veins to the left atrial appendage and finally at the roof of the left atrium for Bachmann’s bundle. The mapping positions are demonstrated on a 3D model in the online Supplementary Video (1). The entire mapping procedure will not prolong the surgical procedure by more than 10–15 min [13].

### Follow-up and Study Endpoint

The postoperative heart rhythm is continuously monitored until hospital discharge and rhythm registrations will be stored in order to determine the incidence of early postoperative AF. After discharge, all patients will be scheduled to visit an out-patient clinic, two times during the first year and thereafter once a year during the following 4 years. Clinical history focused on tachyarrhythmias will be taken and a surface ECG will be made. If indicated, a 24-h Holter recording will be performed. If patients, for any reason, are unable to visit the out-patient clinic, follow-up is done by telephone. In the event that documented rhythm disorders have occurred, records will be requested from the visited hospital. The main endpoint of the study is reached when persistent AF develops.

### Data and Statistical Analysis

**Fig. 2:**
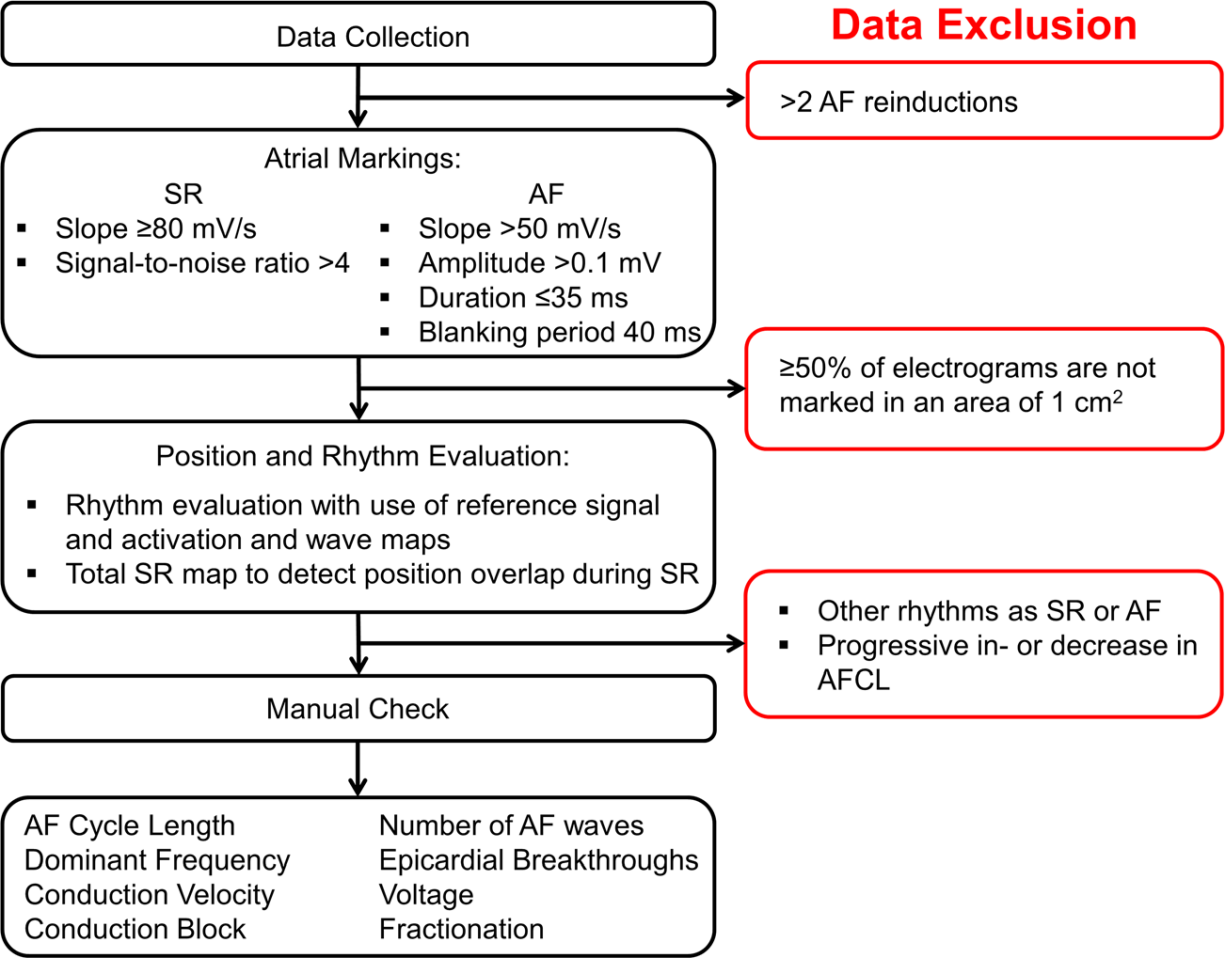
Flow-chart of data evaluation. Atrial fibrillation (*AF*) data from patients that were reinduced >2 times is excluded. Custom-made software detects atrial markings with the presented properties for sinus rhythm (*SR*) and AF. If ≥50 % of a 1 cm^2^ quadrant is not marked, this quadrant will be excluded from further analysis. Rhythm evaluation is performed with use of the activation and wave maps, and the position in SR is evaluated for overlap with a total SR map constructed with use of the reference signal. All data is manually checked, from which the parameters are derived afterwards

**Fig. 3:**
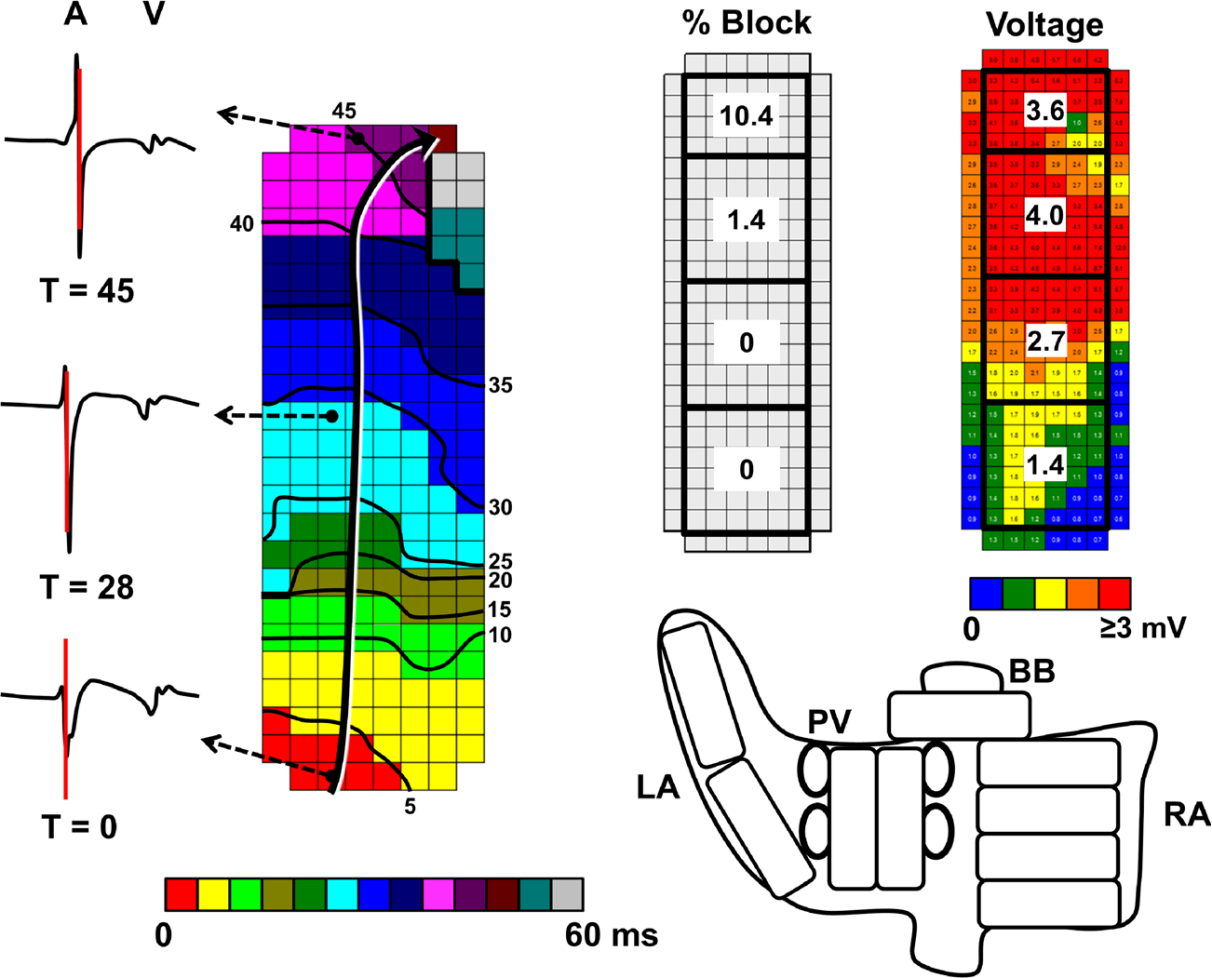
*Left*: activation map constructed during sinus rhythm. The atrial complexes (*A*) of all 192 recordings are automatically detected and marked at the steepest deflection. The electrode with the earliest atrial marking is set at time (*T*) 0. Activation times of the other electrodes are in reference to T_0_. Isochrones are set at 5 ms intervals after T_0_. The *black/white arrow* illustrates the direction of conduction. Conduction block (<18 cm/s) is represented by *thick black lines. V* ventricular complex. *Right*: The mapping surface is divided into quadrants and parameters such as block %, and mean voltage are determined for each quadrant of each mapping location (total: 36 quadrants). *LA* left atrium, *PV* pulmonary veins, *BB* Bachmann’s bundle, *RA* right atrium

**Fig. 4:**
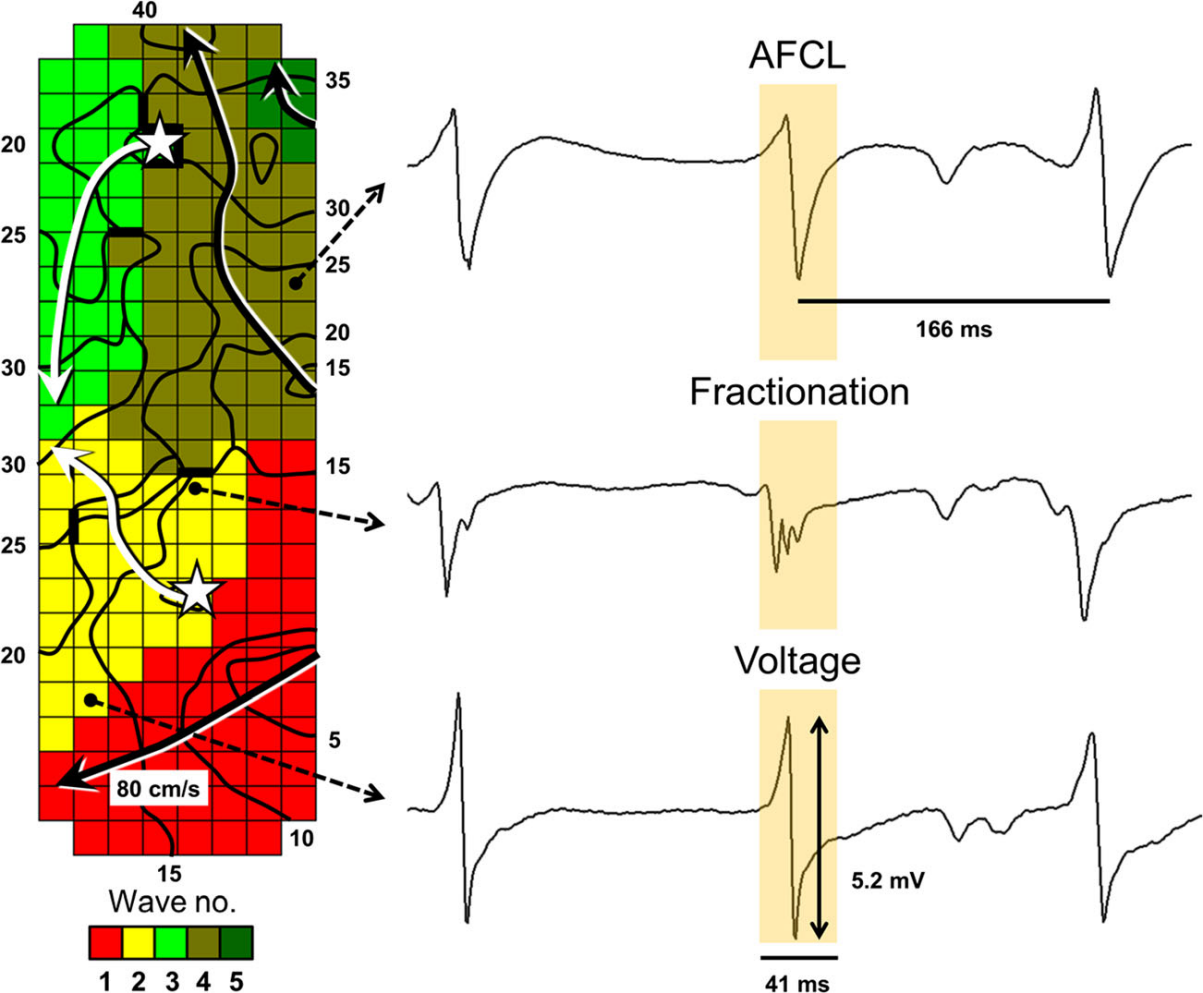
*Left*: wave map during atrial fibrillation at the right atrial free wall. A total of 5 waves activate the recording area in 41 ms; 3 peripheral waves (*black arrows*) and 2 initiate at epicardial breakthroughs (*white star and white arrows*). *Black lines* between electrodes indicate conduction block (<18 cm/s). Isochrones of waves are set at steps of 5 ms after T_0_. Parameters derived from the wavemap include a number of epicardial breakthroughs, waves, and conduction velocity. *Right*: Examples of corresponding electrograms are shown. The parameters that will be derived from electrograms include atrial fibrillation cycle length (AFCL), fractionation, and voltage

Local activation times of the recorded atrial signals will be marked, from which color-coded activation and wave maps will be reconstructed by custom-made software which has previously been described in more detail [11]. Data exclusion criteria include progressive in- or decrease in AF cycle length (AFCL) between sequential recordings (recorded via the reference signal) indicated by an approximately two times in- or decrease in AFCL, recordings of other rhythms than sinus rhythm or AF, or ≥50 % of missing recording data. Data analysis and the criteria for data inclusion are demonstrated in Fig. 2. Electrophysiological parameters that will be derived include conduction velocity, incidence of conduction block, number of fibrillation waves, incidence of epicardial breakthroughs, AFCL, dominant frequency, electrogram voltage (the amplitude of the highest deflection in case of fractionation) and fractionation [11, 12]. For analysis, the electrodes of the mapping array are assigned to quadrants of 1 cm^2^. The variables will consist of averaged values or the percentage of occurrence/incidence for each quadrant. Figure 3 illustrates the construction of an activation map during sinus rhythm, quadrant partition, and its conversion into various parameters of all atrial sites. Figure 4 shows a wave map during AF and the variables that will be analyzed. Furthermore, rotor occurrence and the relation between patterns of activation, fractionation, fibrillation intervals, conduction abnormalities, and voltage will be studied and compared between the different atrial sites, atrial rhythms, and patient groups. Rotors will be defined as a wave of excitation rotating around a phase singularity for one or more cycles [15] and analyzed by determining the dominant frequencies at each recording site in order to identify high-to-low frequency gradients and determination of the degree of linking of fibrillation waves, indicative of repetitive patterns of activation. Linear regression analysis and paired Student’s *t* test will be used to compare various electrophysiological parameters between different sites and different atrial rhythms. Unpaired Student’s *t* test will be used to compare various electrophysiological parameters between patient groups.

The present study is the first that will explore the value of various parameters in discriminating the arrhythmogenic substrate of different patients with AF. We aim for a sample of (at least) 50 subjects in each group for the following reasons. First, our initiative should be considered as an exploratory study. We want to obtain early results in a relative limited number of patients, which will provide a basis for future (in depth) investigations. Therefore, we accept that our study will be underpowered to draw definite conclusions with good precision. Additionally, it is relevant to obtain estimates with sufficient precision, also in early, hypothesis generating studies such as ours. In the binomial distribution, a probability of an observation of 50 % is achieved with the greatest measurement error. Taking that probability as the ‘worst case’, in a dataset of 50 patients, the 95 % confidence intervals (CIs) around an observation would be ±14 %. In the 6*50 = 300 AF patients together, the 95 % CIs would be ±6 %. We consider these precisions acceptable for this exploratory study that will, hopefully, discover parameters that may be used in future studies to discriminate between AF patients with different underlying heart diseases.

### Ethics

The study protocol was approved in February 2010 by the Medical Ethics Committee (2010–054) in the Erasmus Medical Center, Rotterdam, The Netherlands.

## Discussion

### Study Population and Mapping Sites

Previous epicardial mapping studies for AF were performed in small numbers of patients and at only a few atrial sites or with a low resolution [11, 12, 16–19]. The present study is the first to perform intra-operative high-resolution *epicardial mapping* in a large number of patients and enables analyses between patients with different heart diseases. In addition, all sites of both the right and the left atrium accessible from the epicardial side are mapped including Bachmann’s bundle. Bachmann’s bundle might have an important role in the pathophysiology of AF [20]. By mapping the entire surface of both atria there is an increased chance of finding the arrhythmogenic substrate, which might be located in different atrial regions among AF patients.

### The Arrhythmogenic Substrate in AF

The heterogeneous nature in which AF presents and the frequently failing AF treatments so far, demonstrate the importance for an individualized strategy in the treatment of AF. The first step is a better understanding of the pathophysiology underlying initiation and perpetuation of AF. The focus for initiation of AF often originates in the pulmonary veins and Moe et al. described the concept of self-sustaining fibrillatory waves responsible for perpetuation of AF [21, 22]. However, recurrences of persistent AF after successful isolation of the pulmonary veins cannot be explained by these concepts alone. The occurrence of longitudinal dissociation during AF was demonstrated later on and showed to be most prominent in persistent AF [11]. Furthermore, focal fibrillation waves emerging within the recording area, referred to as epicardial breakthroughs, occur much more frequently during persistent AF than during acute AF [12], as well as drivers such as rotors and focal sources [23]. These findings suggest that progressive electro-pathological changes within the atria are associated with persistent AF. Nonetheless, the exact pathophysiological changes and locations at which they occur are not yet known. The underlying diseases most likely initiate different pathophysiological mechanisms that lead to AF. For example, valvular disorders give rise to atrial pressure or volume overload, coronary artery disease can cause atrial ischemia and infarction, and congenital heart diseases may also include congenital atrial abnormalities. For this reason, the patients in this study are divided in separate groups according to the underlying heart disorders and AF occurrence prior to surgery.

Previous studies have investigated the underlying cause responsible for perpetuation of AF. Atrial fibrosis has been suggested to be an important element in the pathophysiology of AF. There is a significant larger amount of atrial fibrosis seen in patients with AF [7, 24]. An excessive extracellular matrix leads to uncoupling of cells and may facilitate inhomogeneous conduction, re-entry, and multiple wavelets. MRI or electro-anatomical voltage mapping can be helpful diagnostic tools for the determination of degree of fibrosis in AF patients and identification of areas of fibrosis. However, no association has been found between the amount of fibrosis and clinical AF characteristics [7, 24]. Electrical signal conduction involves processes on a structural, cellular and molecular level, and these together determine if conduction is altered and AF occurs. Therefore, the arrhythmogenic substrate can probably be more accurately localized by measuring electrical potentials and conduction. In the present study, both the recorded extracellular potentials and the spatial domain of the electrograms enables conversion into specific electrophysiological parameters that could identify areas with conduction abnormalities. If proven successful, this strategy can be developed into a diagnostic tool for each individual AF patient. In addition, current ablation strategies aimed at identifying and targeting arrhythmogenic areas are not effective in a large proportion of patients and might even lead to new arrhythmias [25]. If patients that can benefit could be selected beforehand, effectiveness of these treatments might improve.

### Study Limitations

Currently, epicardial mapping can only be performed during open-chest cardiac surgery. Therefore, it is not possible to perform epicardial mapping in patients with nondiseased hearts. However, with constantly advancing techniques it may become possible in the future to perform epicardial mappings during video-assisted thoracoscopic surgery in patients without any heart disease. Secondly, although epicardial mapping can reach sites endocardial mapping cannot, some sites are not accessible, for example, the atrial septum. Therefore, epicardial mapping is not able to analyze conduction in the entire area of the atria. In addition, recordings are performed sequentially, as simultaneous high-resolution mapping of the entire surface is not possible yet with currently available technical equipment. As time during surgery is limited, mapping is performed immediately after AF induction or electrical conversion. Consequently, if a progressive increase, or decrease in AFCL occurs during the recordings, this data will have to be excluded [26–28]. General anesthesia may also increase AFCL [29]. However, the same anesthetic protocol is applied in all patients and previous studies have shown that there remain differences in AF between patients despite anesthesia [11, 12]. Furthermore, recent studies have shown that endo-epicardial dissociation can occur during AF and might be associated with persistent AF [30]. This suggests that it is important to investigate endocardial and epicardial conduction simultaneously as conduction can be disturbed in all three dimensions. Finally, there is a small chance asymptomatic persistent AF episodes may be undetected during follow-up. The measured incidence of persistent late postoperative AF may therefore underestimate the true incidence of persistent late postoperative AF.

### Clinical Relevance

This project can provide the tools to discriminate the arrhythmogenic substrate of AF in patients with different heart diseases and is potentially the first step towards a patient-tailored strategy for the treatment of AF.

### Project Status

At present, the inclusion for this study is ongoing. We expect the data of this project to become available in 2016 or 2017.

## Electronic Supplementary Material

Below is the link to the electronic supplementary material.

Supplementary video 1 Mapping positions of the rectangular 192-electrode array on a 3D model of the heart (MP4 58077 kb)

## Abbreviations

AF: Atrial fibrillation
CABG: Coronary artery bypass grafting
ECG: Electrocardiogram
AFCL: Atrial fibrillation cycle length

## References

[1] Gaita F, Caponi D, Scaglione M (2008). Long-term clinical results of 2 different ablation strategies in patients with paroxysmal and persistent atrial fibrillation. Circulation. Arrhythmia and Electrophysiology.

[2] Ganesan AN, Shipp NJ, Brooks AG (2013). Long-term outcomes of catheter ablation of atrial fibrillation: a systematic review and meta-analysis. Journal of the American Heart Association.

[3] Mulder AA, Wijffels MC, Wever EF, Boersma LV (2012). Freedom from paroxysmal atrial fibrillation after successful pulmonary vein isolation with pulmonary vein ablation catheter-phased radiofrequency energy: 2-year follow-up and predictors of failure. Europace.

[4] Lafuente-Lafuente C, Valembois L, Bergmann JF, Belmin J (2015). Antiarrhythmics for maintaining sinus rhythm after cardioversion of atrial fibrillation. Cochrane Database of Systematic Reviews.

[5] Kannel WB, Wolf PA, Benjamin EJ, Levy D (1998). Prevalence, incidence, prognosis, and predisposing conditions for atrial fibrillation: population-based estimates. The American Journal of Cardiology.

[6] Psaty BM, Manolio TA, Kuller LH (1997). Incidence of and risk factors for atrial fibrillation in older adults. Circulation.

[7] Kottkamp H (2013). Human atrial fibrillation substrate: towards a specific fibrotic atrial cardiomyopathy. European Heart Journal.

[8] Nademanee K, McKenzie J, Kosar E (2004). A new approach for catheter ablation of atrial fibrillation: mapping of the electrophysiologic substrate. Journal of the American College of Cardiology.

[9] Narayan SM, Krummen DE, Shivkumar K, Clopton P, Rappel WJ, Miller JM (2012). Treatment of atrial fibrillation by the ablation of localized sources: CONFIRM (Conventional Ablation for Atrial Fibrillation With or Without Focal Impulse and Rotor Modulation) trial. Journal of the American College of Cardiology.

[10] Scherlag BJ, Nakagawa H, Jackman WM, Yamanashi WS, Patterson E, Po S, Lazzara R (2005). Electrical stimulation to identify neural elements on the heart: their role in atrial fibrillation. Journal of Interventional Cardiac Electrophysiology.

[11] Allessie MA, de Groot NM, Houben RP, Schotten U, Boersma E, Smeets JL, Crijns HJ (2010). Electropathological substrate of long-standing persistent atrial fibrillation in patients with structural heart disease: longitudinal dissociation. Circulation. Arrhythmia and Electrophysiology.

[12] de Groot NM, Houben RP, Smeets JL (2010). Electropathological substrate of longstanding persistent atrial fibrillation in patients with structural heart disease: epicardial breakthrough. Circulation.

[13] Yaksh A, van der Does LJ, Kik C (2015). A novel intra-operative, high-resolution atrial mapping approach. Journal of Interventional Cardiac Electrophysiology.

[14] Yaksh A, Kik C, Knops P (2014). Atrial fibrillation: to map or not to map?. Netherlands Heart Journal.

[15] Chen J, Mandapati R, Berenfeld O, Skanes AC, Gray RA, Jalife J (2000). Dynamics of wavelets and their role in atrial fibrillation in the isolated sheep heart. Cardiovascular Research.

[16] Kanagaratnam P, Kojodjojo P, Peters NS (2008). Electrophysiological abnormalities occur prior to the development of clinical episodes of atrial fibrillation: observations from human epicardial mapping. Pacing and Clinical Electrophysiology.

[17] Lee G, Kumar S, Teh A (2014). Epicardial wave mapping in human long-lasting persistent atrial fibrillation: transient rotational circuits, complex wavefronts, and disorganized activity. European Heart Journal.

[18] Nitta T, Ishii Y, Miyagi Y, Ohmori H, Sakamoto S, Tanaka S (2004). Concurrent multiple left atrial focal activations with fibrillatory conduction and right atrial focal or reentrant activation as the mechanism in atrial fibrillation. The Journal of Thoracic and Cardiovascular Surgery.

[19] Sueda T, Nagata H, Shikata H (1996). Simple left atrial procedure for chronic atrial fibrillation associated with mitral valve disease. The Annals of Thoracic Surgery.

[20] van Campenhout MJ, Yaksh A, Kik C, de Jaegere PP, Ho S, Allessie MA, de Groot NM (2013). Bachmann’s bundle: a key player in the development of atrial fibrillation?. Circulation. Arrhythmia and Electrophysiology.

[21] Haissaguerre M, Jais P, Shah DC (1998). Spontaneous initiation of atrial fibrillation by ectopic beats originating in the pulmonary veins. The New England Journal of Medicine.

[22] Moe GK, Abildskov JA (1959). Atrial fibrillation as a self-sustaining arrhythmia independent of focal discharge. American Heart Journal.

[23] Baykaner T, Lalani GG, Schricker A, Krummun DE, Narayen SM (2014). Mapping and ablating stable sources for atrial fibrillation: summary of the literature on Focal Impulse and Rotor Modulation (FIRM). Journal of Interventional Cardiac Electrophysiology.

[24] Boldt A, Wetzel U, Lauschke J (2004). Fibrosis in left atrial tissue of patients with atrial fibrillation with and without underlying mitral valve disease. Heart.

[25] Wu SH, Jiang WF, Gu J (2013). Benefits and risks of additional ablation of complex fractionated atrial electrograms for patients with atrial fibrillation: a systematic review and meta-analysis. International Journal of Cardiology.

[26] Ravelli F, Mase M, Del Greco M, Faes L, Disertori M (2007). Deterioration of organization in the first minutes of atrial fibrillation: a beat-to-beat analysis of cycle length and wave similarity. Journal of Cardiovascular Electrophysiology.

[27] Haissaguerre M, Sanders P, Hocini M (2004). Changes in atrial fibrillation cycle length and inducibility during catheter ablation and their relation to outcome. Circulation.

[28] Roithinger FX, Karch MR, Steiner PR, SippensGroenewegen A, Lesh MD (1997). Relationship between atrial fibrillation and typical atrial flutter in humans: activation sequence changes during spontaneous conversion. Circulation.

[29] Holm M, Johansson R, Smideberg B, Lührs C, Olsson SB (1999). Effect of cardiac exposure by median sternotomy on atrial fibrillation cycle length. Europace.

[30] Eckstein J, Zeemering S, Linz D (2013). Transmural conduction is the predominant mechanism of breakthrough during atrial fibrillation: evidence from simultaneous endo-epicardial high-density activation mapping. Circulation. Arrhythmia and Electrophysiology.

